# Supplementary material for: The methyl-CpG-binding protein 2 inhibits cGAS-associated signaling
Source: Nat Commun. 2025 Nov 7;16:9827. doi: 10.1038/s41467-025-65713-z (PMC12595089; doi:10.1038/s41467-025-65713-z)
Supplement: Supplementary file 1 — Supplementary Information File [file 41467_2025_65713_MOESM1_ESM.pdf]

TITLE

The methyl-CpG-binding protein 2 inhibits cGAS-associated signaling

Supplementary Figures Fig. 1 –6

Source data for Western blots of supplementary Figures.

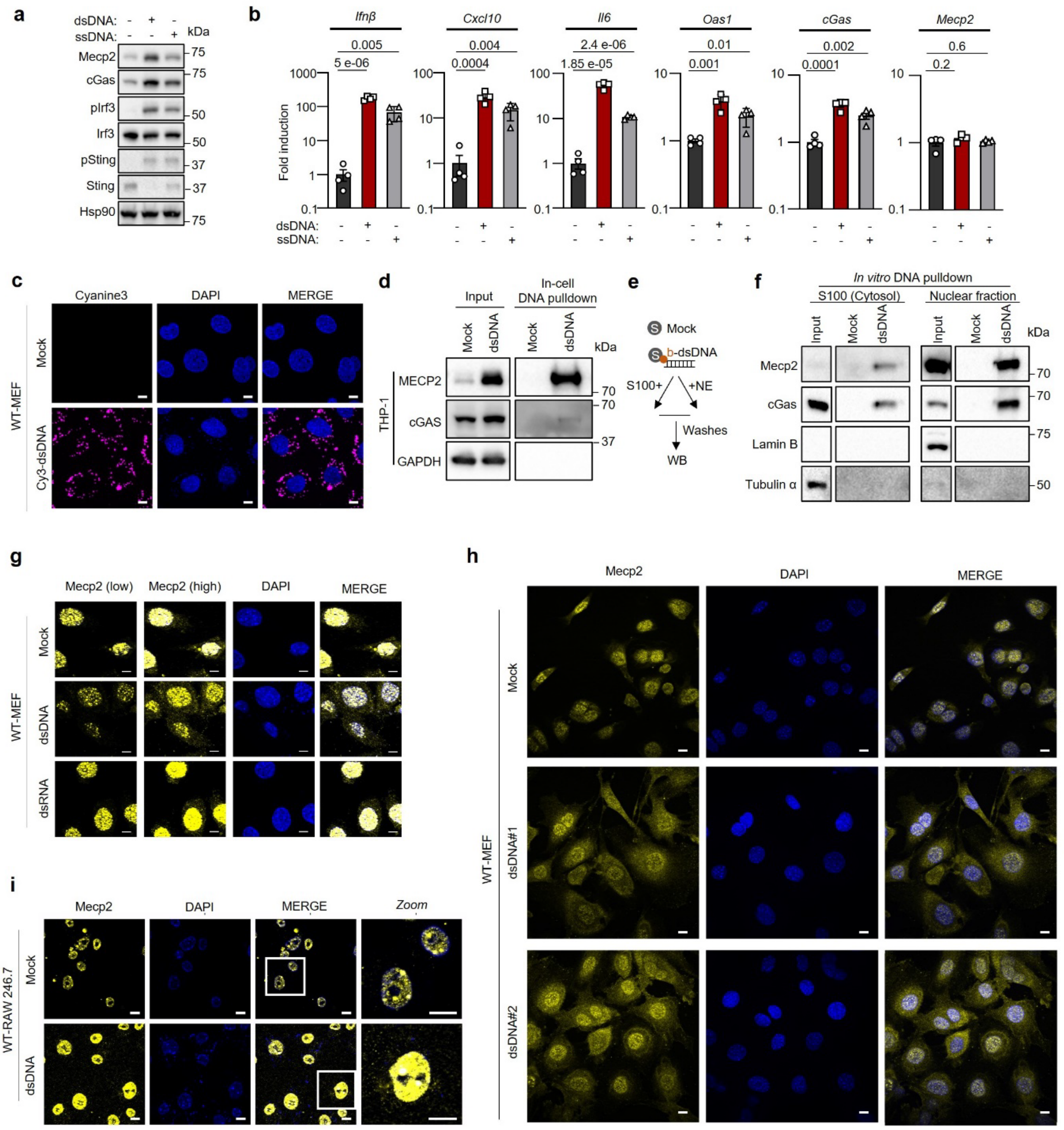

Supplementary Figure 1. **MeCP2 interacts with cytosolic dsDNA**. **a** Whole cell extracts (WCE) prepared from WT-MEF transfected or not with dsDNA or ssDNA for 6 hours were analyzed by western blot (WB) using indicated antibodies. **b** *Ifnβ*, *Cxcl10*, *Il6*, *Oas1, cGas* and *Mecp2* mRNA levels were analyzed in WT-MEF treated as in **a**. Graphs present the mean ± standard deviation of mean (SEM) from 4 independent experiments. **c** Imaging of WT-MEF cells transfected or not for 6 hours with Cy3-dsDNA and DAPI nuclear staining. Scale bar: 10µm. Images are representative of 3 independent experiments. **d** THP-1 were transfected or not with biotinylated b-dsDNA before whole-cell extract preparation and pull-down using streptavidin-affinity beads. Input and eluates were analyzed by WB using the indicated antibodies. **e** Experimental scheme for f. **f** Cytosolic (S100) and nuclear (NE) fractions prepared from WT-MEF, were incubated with streptavidin beads alone or with streptavidin bead-bound b-dsDNA prior to pulldown. Input and eluates were analyzed by WB using the indicated antibodies. **g** Immunofluorescence analyses were performed on WT-MEF transfected with dsDNA or dsDNA using anti-MeCP2 antibody and DAPI nuclear staining. Scale bar 10µm. Images are representative of 2 independent experiments. **h** WT-MEFs were transfected or not

with dsDNAs with different sequences prior to immunofluorescence analysis using anti-MeCP2 antibody and DAPI nuclear staining. **i** Immunofluorescence analysis was performed on WT-RAW264.7 transfected or not with dsDNA using anti-MeCP2 antibody and DAPI nuclear staining. Scale bar: 10µm. Images are representative of at least 3 independent experiments. WB are representative of 3 independent experiments. Significance was assessed using two-sided Student T-test. Source data are provided as a Source Data file for numerical data and at the end of the Supplementary Information File for Western blots.

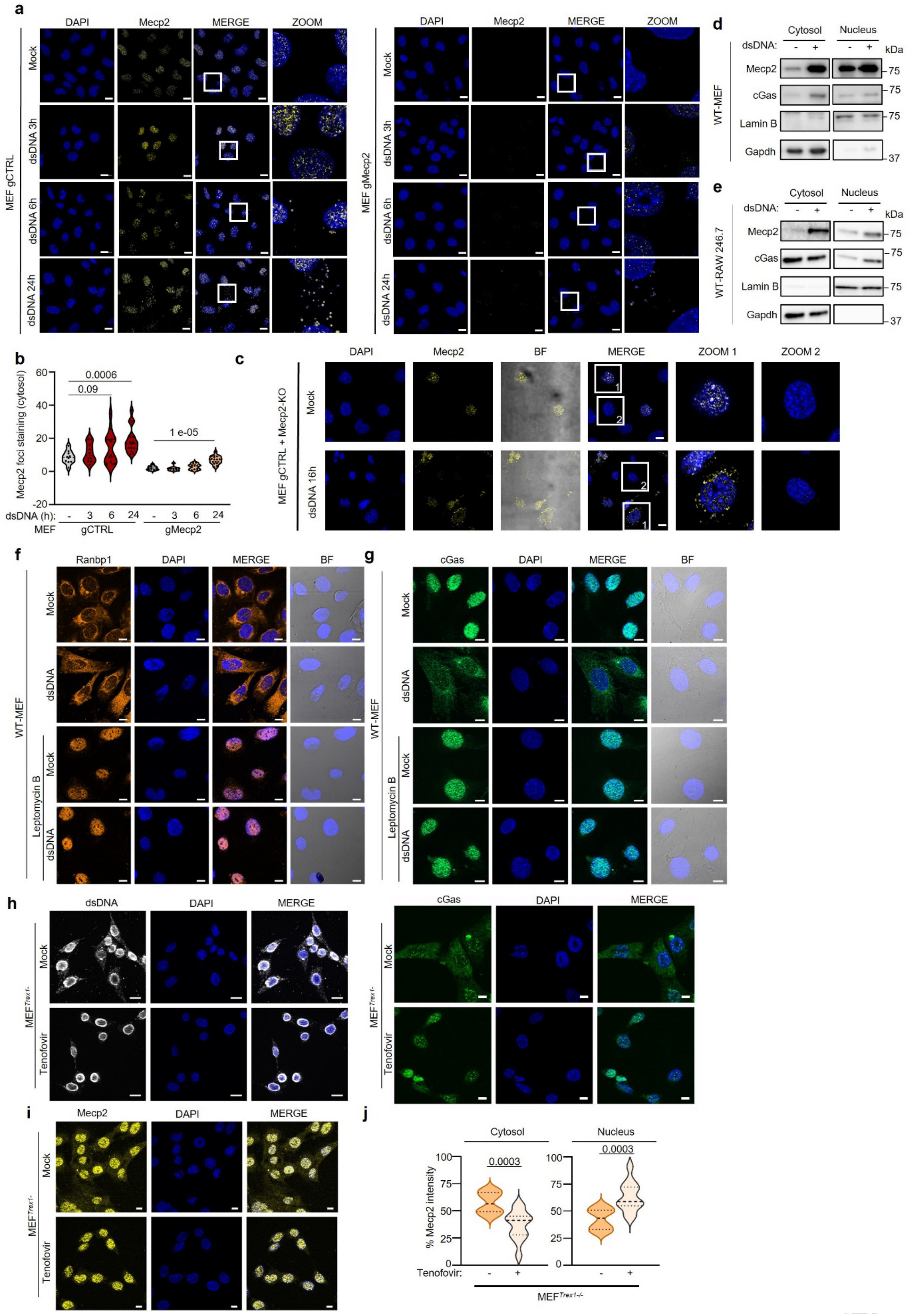

Supplementary Figure 2. **dsDNA challenge triggers MeCP2 export. a** MEF^{gCTRL} or MEF^{gMecp2} were transfected or not with dsDNA for 3, 6 and 24h prior to immunofluorescence analysis using an anti-MeCP2 antibody and DAPI nuclear staining. Images are representative

of 2 independent experiments. Scale bar: 10µm. **b** Quantification of Mecp2 cytosolic foci in images acquired as in a. c. MEF^{gMecp2} and MEF^{gCTRL} were mixed (1:1 ratio) prior to transfection for 16 hours with dsDNA. Immunofluorescence analysis was conducted using an anti-MeCP2 antibody and DAPI nuclear staining. Images are representative of 2 independent experiments. Scale bar: 10µm. **d** Cytosolic and nuclear extracts were prepared from WT-MEF transfected or not with dsDNA for 6 hours. Fractions were analyzed by WB using indicated antibodies. WB are representative of at least 3 independent experiments. **e** Cytosolic and nuclear extracts were prepared from WT-RAW264.7 transfected or not with dsDNA for 6 hours. Fractions were analyzed by WB using indicated antibodies. WB are representative of at least 3 independent experiments. **f** Immunofluorescence analysis was performed on WT-MEF treated or not with 20 nM of Leptomycin B for 1 hour prior to dsDNA transfection for 3 hours, using an anti-Ranbp1 antibody and DAPI nuclear staining. Scale bar: 10µm. Images are representative of two independent experiments. **g** Immunofluorescence was performed on WT-MEFs treated as in **f** except that an anti-cGas antibody was used. **h** Immunofluorescence analysis was performed on $MEF^{Trex1-/-}$ treated or not with tenofovir, using anti-dsDNA or anti-cGas antibodies, and DAPI nuclear staining. Scale bar: 20 µm. Images are representative of two independent experiments. **i** As in **h**, except that an anti-MeCP2 antibody was used. Scale bar: 10 µm. Images are representative of two independent experiments. **j** Violin plots show the % of MeCP2 intensity in the cytosol and in the nucleus; n=12 cells per condition. Significance was assessed using two-sided Student T-test. Source data are provided as a Source Data file for numerical data and at the end of the Supplementary Information File for Western blots.

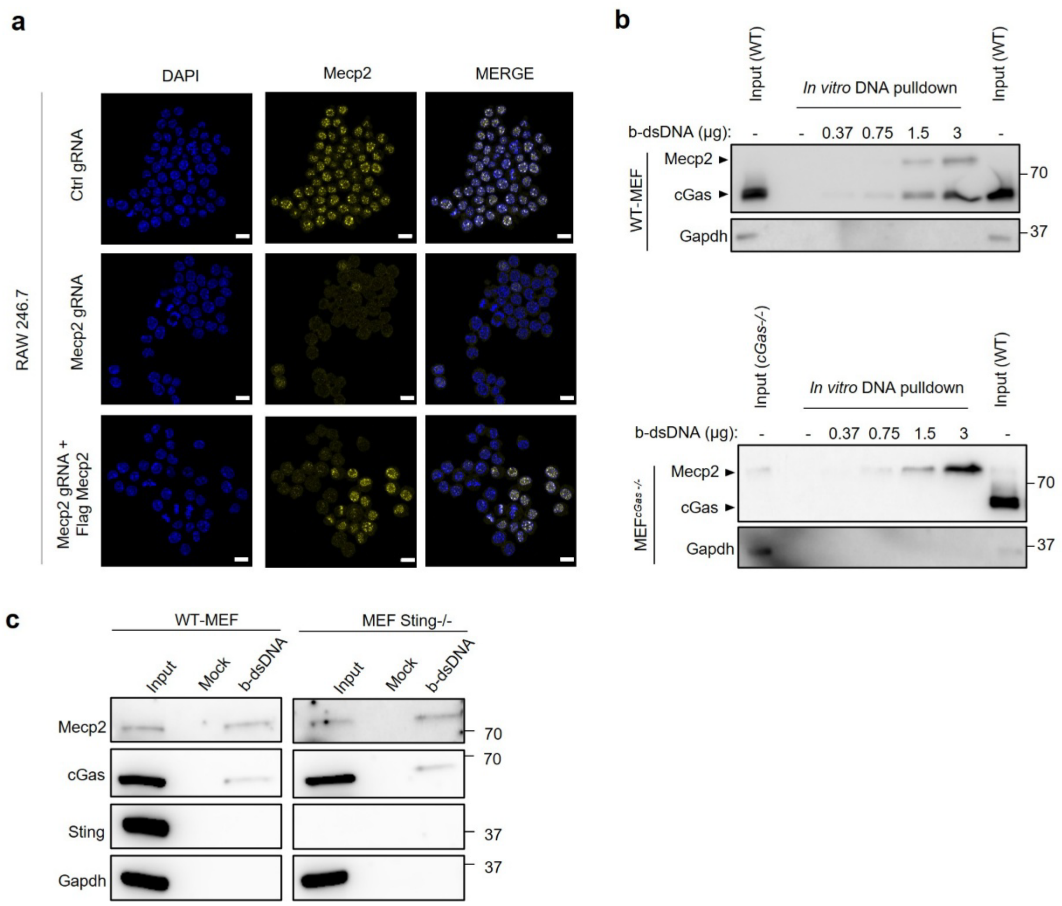

Supplementary Figure 3. **Absence of cGas promotes enhanced MeCP2 interaction with dsDNA. a** Immunofluorescence was conducted using an anti-Mecp2 antibody and DAPI nuclear staining on RAW264.7^{gCTRL} RAW264.7^{gMecp2} as well as RAW264.7^{gMecp2} stably expressing a gRNA resistant FLAG-MeCP2 construct. Representative Image. Scale bar: 10µm. **b** Whole cell extracts prepared from WT-MEF (left) or MEF$^{cGas-/-}$ (right) were incubated with increasing quantity of streptavidin bead-bound b-dsDNA (as indicated). Input and eluates were analyzed by WB using the indicated antibodies. **c** *In vitro* pulldowns were performed using whole cell extracts prepared from WT-MEF or Sting knockout MEF (MEF$^{Sting-/-}$) using beads-bound biotinylated dsDNA Input and eluates were analyzed by Western blot (WB) using the indicated antibodies. Source data are provided as a Source Data file for numerical data and at the end of the Supplementary Information File for Western blots.

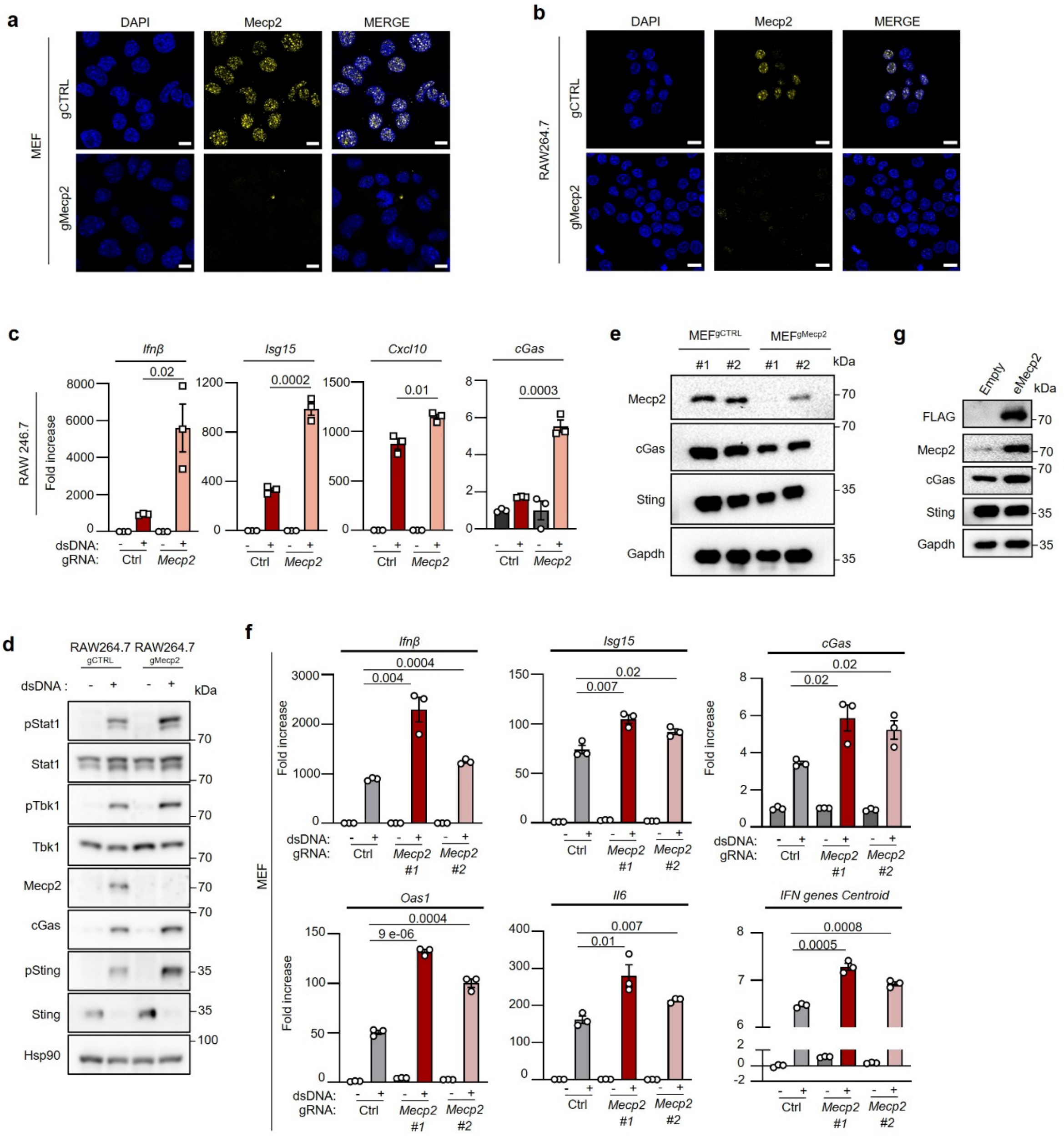

Supplementary Figure 4. **Absence of MeCP2 enhances cGas-Sting activation. a** MEF^{gCTRL} and MEF^{gMecp2} were subjected to immunofluorescence analysis using anti-MeCP2 antibody and DAPI nuclear staining. Images are representative of 3 independent immunostainings. Scale bar: 10µm. **b** RAW264.7^{gCTRL} and RAW264.7^{gMecp2} were subjected to immunofluorescence analysis using anti-MeCP2 antibody and DAPI nuclear staining. Images are representative of 3 independent immunostainings. Scale bar: 10µm. **c** RAW264.7^{gCTRL} and RAW264.7^{gMecp2} were challenged or not with dsDNA for 6 hours prior to gene expression analysis. Graphs present mean (± SEM) *Ifnβ*, *Isg15*, *Cxcl10* and *cGas* mRNA levels (n=3 independent experiments). **d** WB analyses were performed on cells treated as in **c** using indicated antibodies. **e** WB was conducted on MEF cells expressing two different control gRNAs and 2 different MeCP2 targeting gRNAs. WB is representative of 2 different immunostaining experiments. **f** Cells expressing 2 different gRNAs were transfected or not with dsDNA prior to gene expression analyses. Graphs present mean (± SEM) *Ifnβ*, *Isg15*, *cGas*, *Il6* and *Oas1* mRNA levels and mean centroid analysis (n=3 independent experiments). **g** WT-MEF expressing FLAG tagged MeCP2 were analyzed by WB using the indicated antibodies. WB are representative of 3

independent experiments. Significance was assessed using two-sided Student T-test. Source data are provided as a Source Data file for numerical data and at the end of the Supplementary Information File for Western blots.

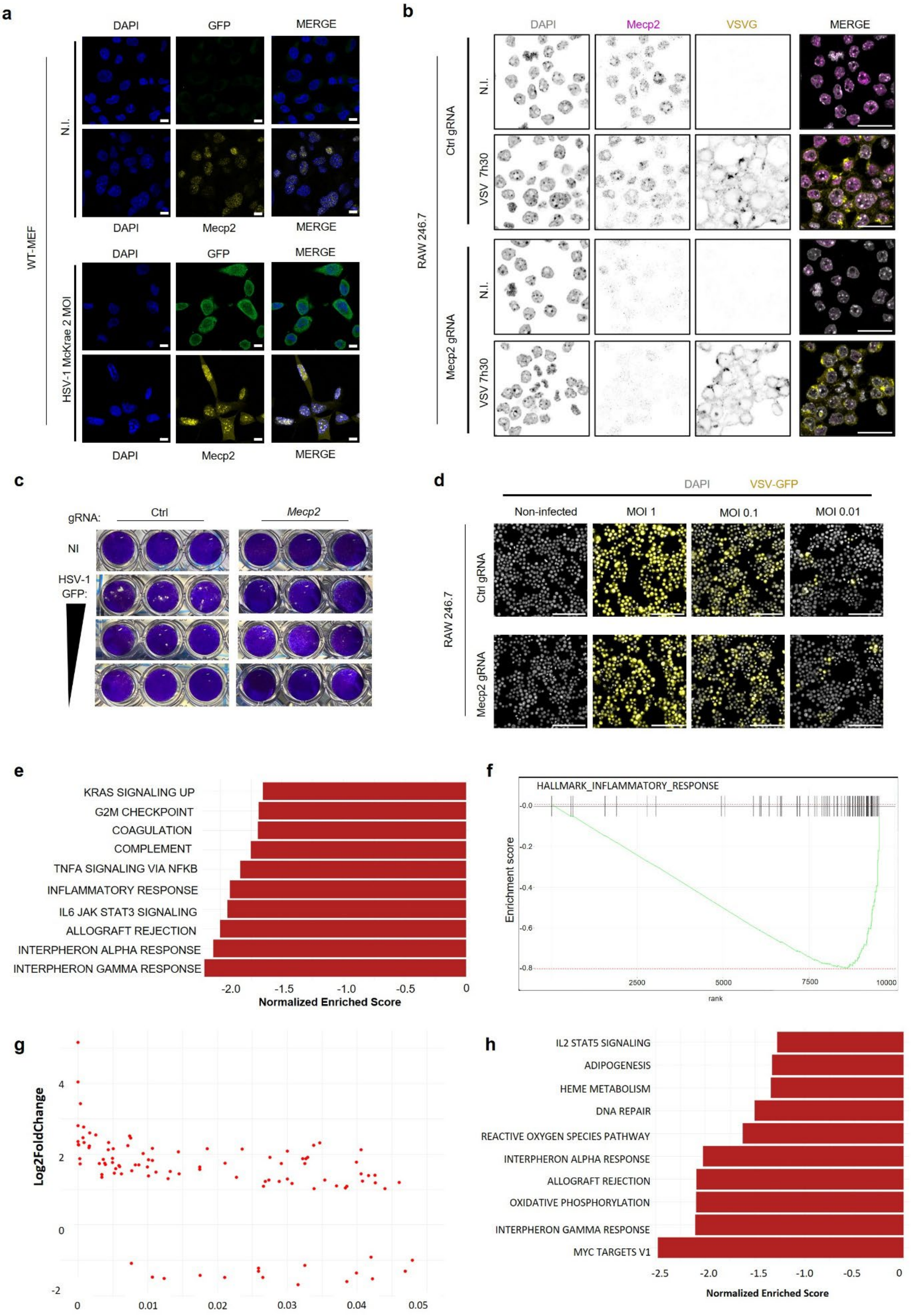

Supplementary Figure 5. **Absence of MeCP2 enforces an antiviral state. a** WT-MEF were infected or not with GFP-expressing HSV-1 McKrae prior to immunostaining using MeCP2-

and GFP-specific antibodies and DAPI nuclear staining. Images are representative of 3 independent experiments. Scale bar: 10µm. **b** RAW264.7^{gCTRL} or RAW264.7^{gMecp2} were infected or not with VSV for 7.5 hours prior to immunofluorescence analysis using anti-MeCP2 and VSV-G specific antibodies and DAPI nuclear staining. Images are representative of 2 independent experiments. Scale bar: 20µm. **c** Example images of plaques quantified in Figure 5F. **d** As in **b**, except that cells were infected with a range of VSV-G MOIs and GFP signal, attesting to VSV infection is shown. Images are representative of 2 independent experiments. Scale bar: 100µm. **e** Top10 Gene Sets upregulated in mice with non-functioning MeCP2 versus controls. **f** The gene set "Inflammatory Response" is reported as an example of one Gene Set upregulated in mice with no MeCP2 protein versus controls. **g** Patients with RTT with different severity of symptoms. The plot reports the significant DEGs that are involved in inflammation, interferon-beta, virus response, STING and innate immunity among the DEGs between patients with RTT with mild symptoms and patients of RTT with moderate to severe respiratory phenotype. Y axis: log2FoldChange, the higher the log2FoldChange, the higher in the expression in samples derived from patients with mild symptoms. X axis: p-value adjusted (False Discovery Rate-FDR). **h** Top10 Gene Sets upregulated in patients with RTT with moderate to severe symptoms versus patients with mild symptoms. Source data are provided in the Source Data file.

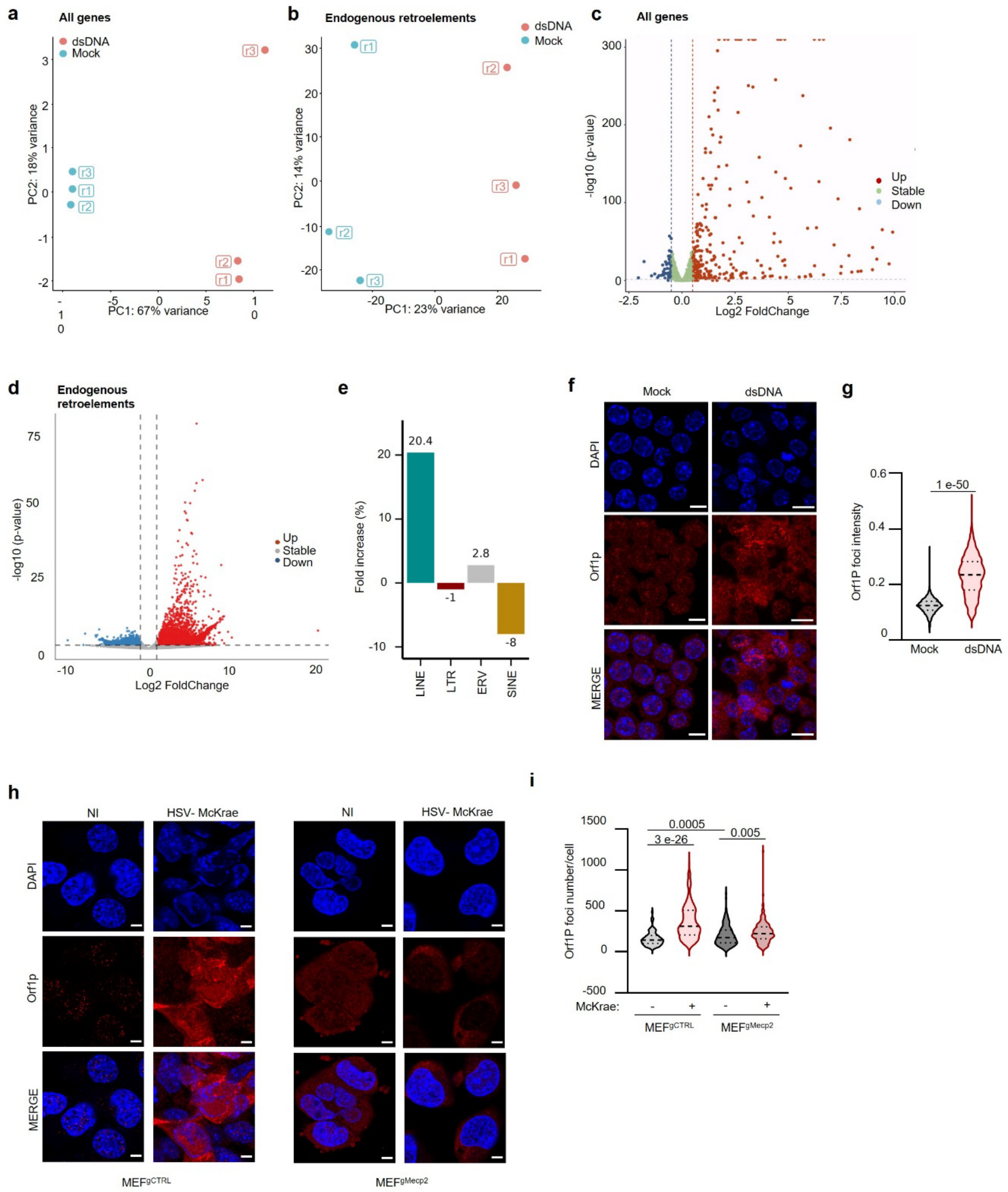

Supplementary Figure 6. **MeCP2 deficiency leads to accumulation of immunogenic LINE-1 derived DNA. a** Principal Component Analysis (PCA) plot of all transcripts identified in RNAseq analyses conducted on RAW267.4 cells transfected of not with dsDNA for 6 hours. **b** PCA plot of transcripts corresponding to endogenous retroelements in samples from **a**. **c** Volcano plot representing upregulated, stable or downregulated transcripts in samples from **a**. **d** Volcano plot representing upregulated, stable or downregulated transcripts corresponding to endogenous retroelements in WT-MEF stimulated with dsDNA for 6 hours as compared to non-stimulated cells. **e** Graph presents the % fold increase in transcripts corresponding to type I endogenous retroelements in data from **d**. LINE: long interspersed nuclear elements, LTR: Long terminal repeats containing retroelements; ERV: Endogenous RetroViruses; SINE: short interspersed repetitive elements. **f** Immunofluorescence analysis of WT-MEF transfected or not

with dsDNA for 6 hours using anti-Orf1p antibody and DAPI nuclear staining. Scale bar: 10µm. Images are representative of 3 independent experiments. **g** Graph presents the mean Orf1p signal intensity quantified in images acquires as in **f**. **h** Immunofluorescence analysis of WT-MEF infected or not with GFP-expressing HSV-1 McKrae for 16 hours using anti-Orf1p antibody and DAPI nuclear staining. Scale bar: 10µm. Images are representative of 3 independent experiments. **i** Graphs present the mean Orf1p foci number per cell in images acquired as in **h;** n>150 cells per condition. Significance was assessed using two-sided Student T-test. Source data are provided in the Source Data file.

RAW data for Supplementary figures

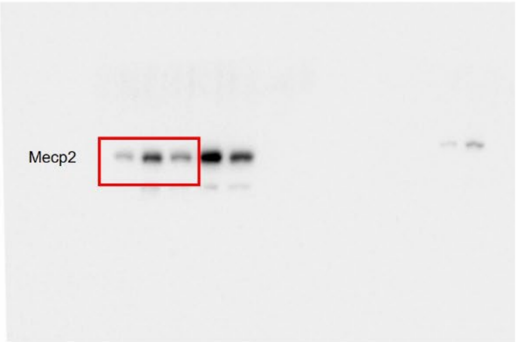

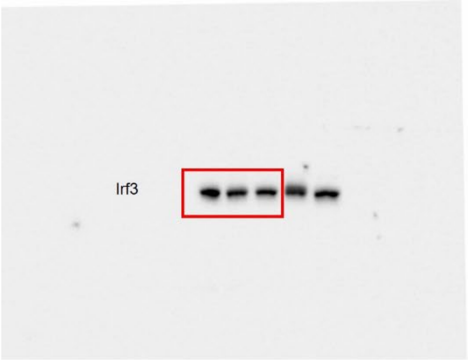

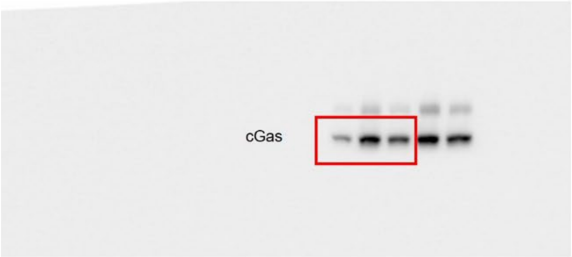

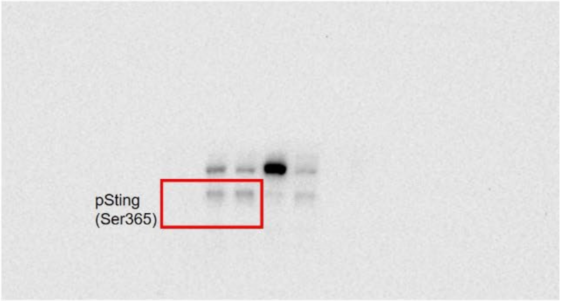

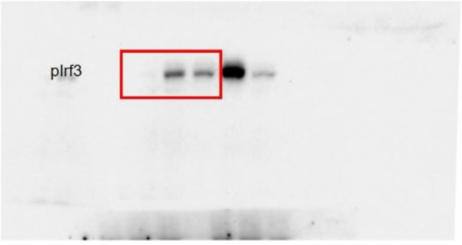

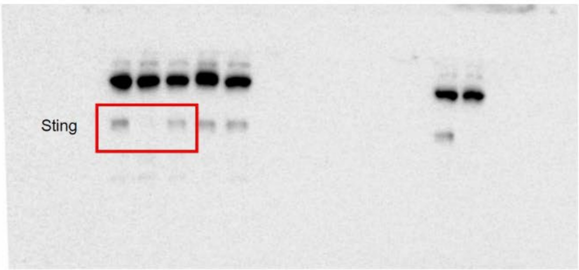

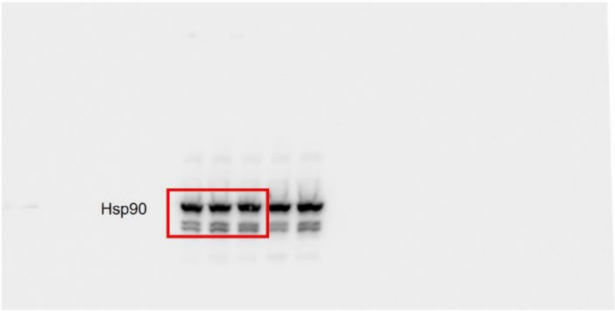

RAW data for Supplementary figure 1A.

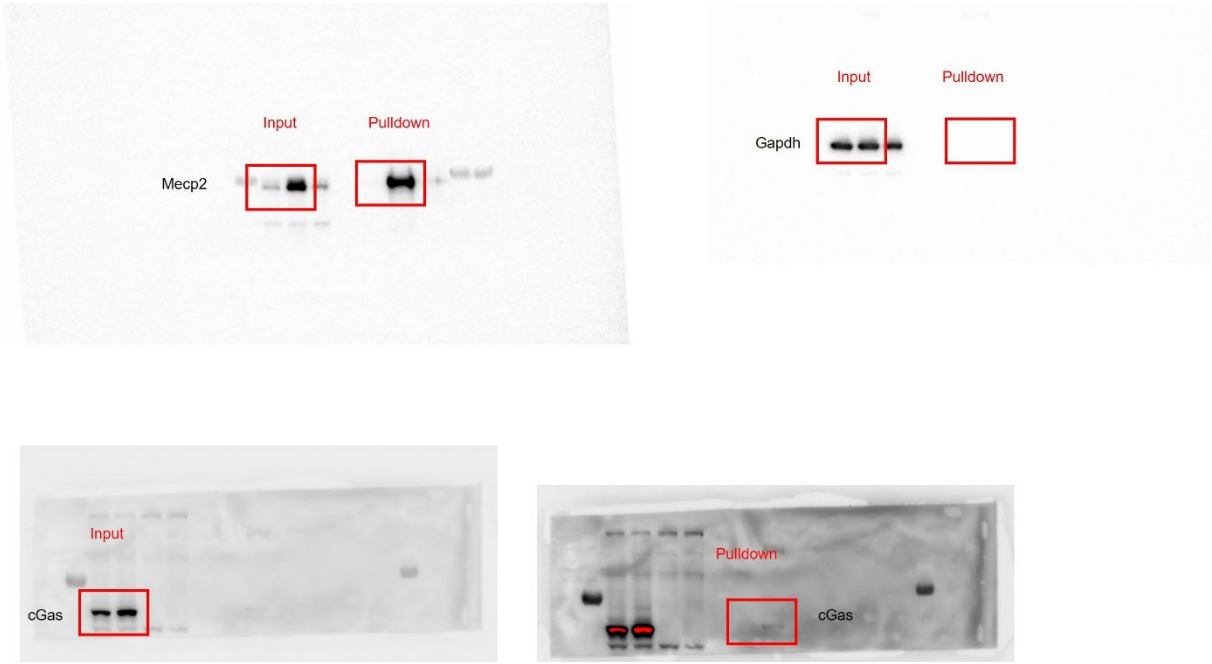

RAW data for Supplementary figure 1D.

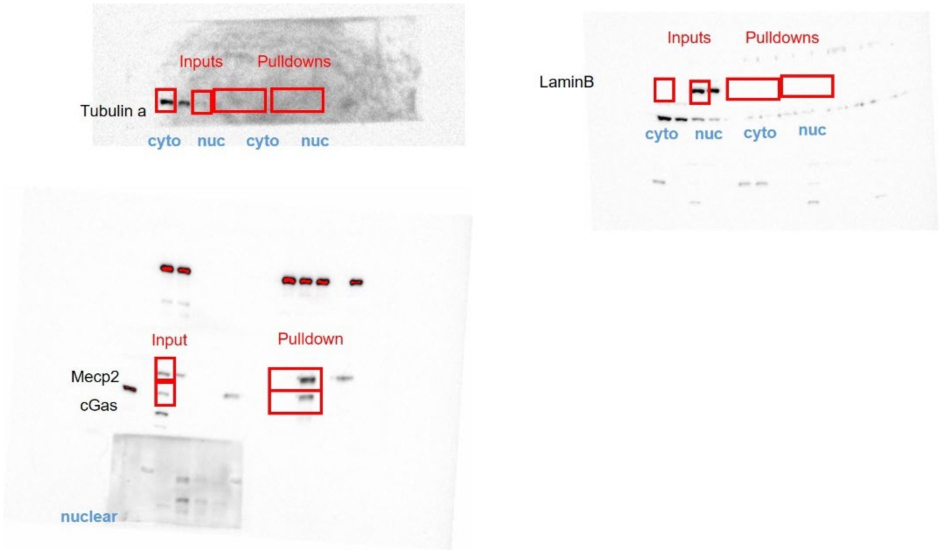

RAW data for Supplementary figure 1F.

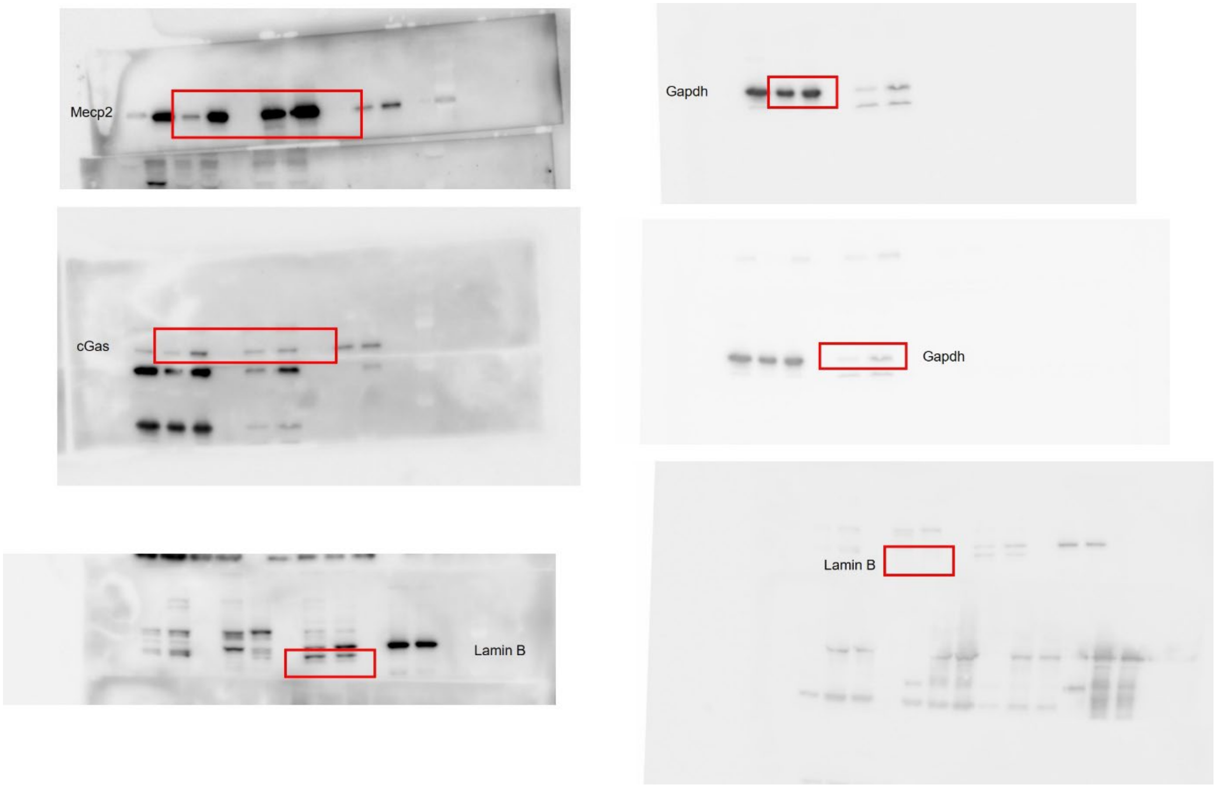

RAW data for Supplementary figure 2A.

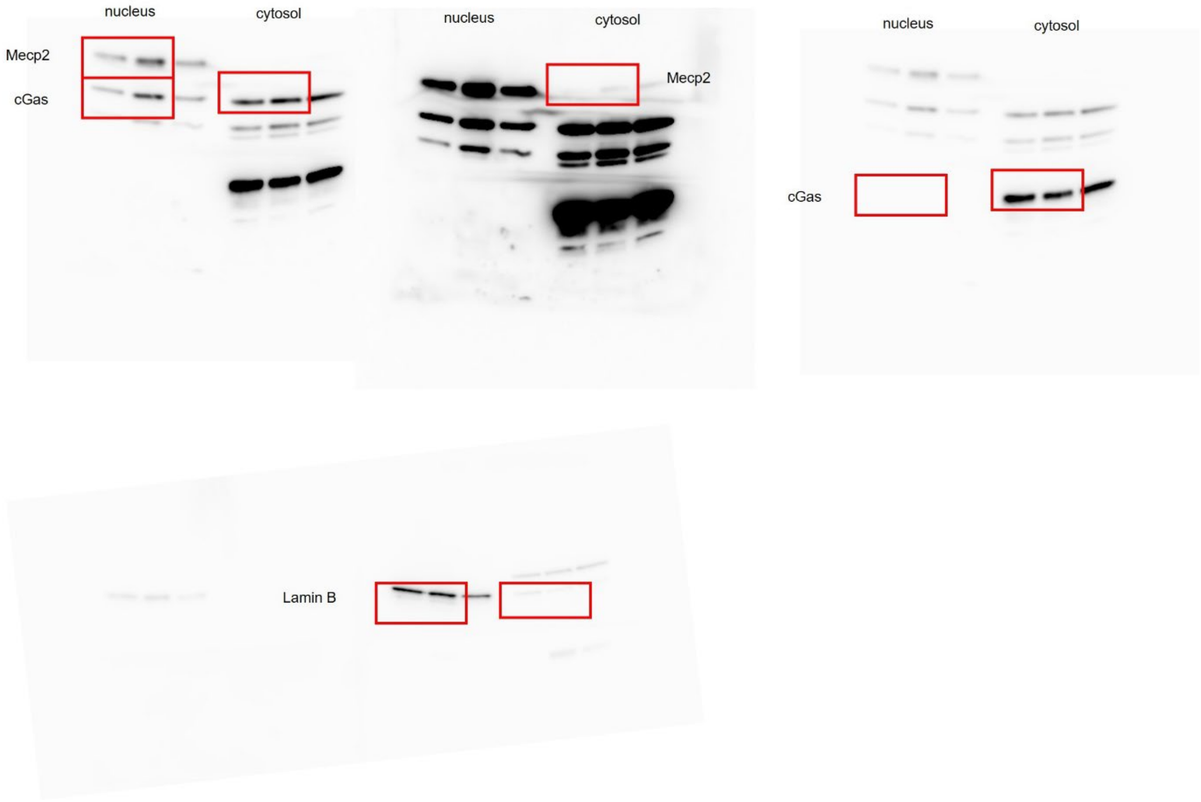

RAW data for Supplementary figure 2B.

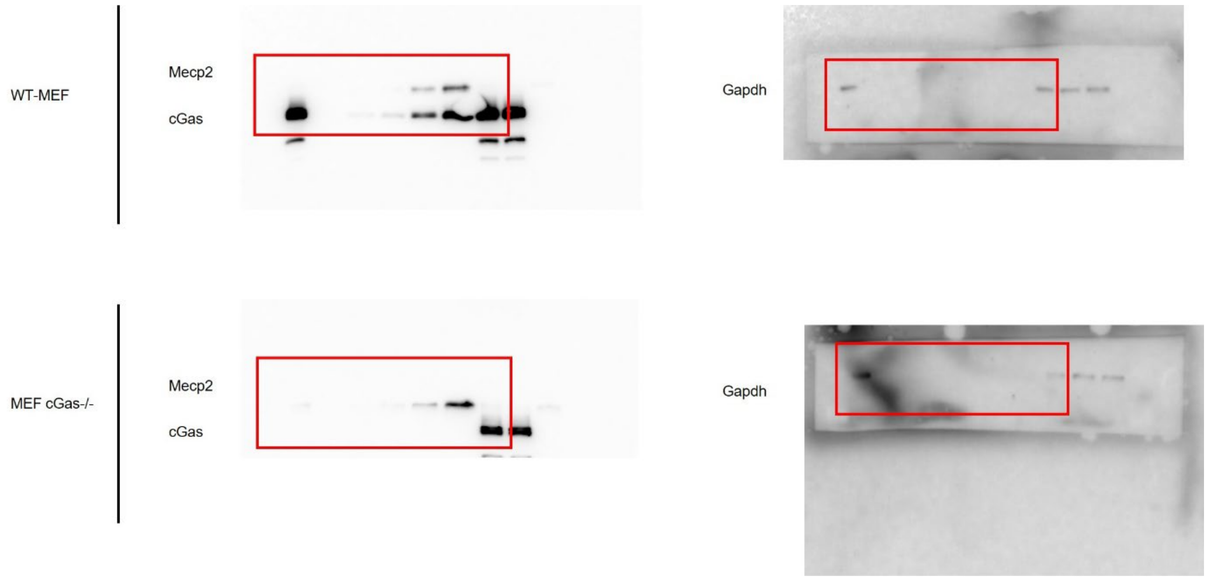

RAW data for Supplementary figure 3B.

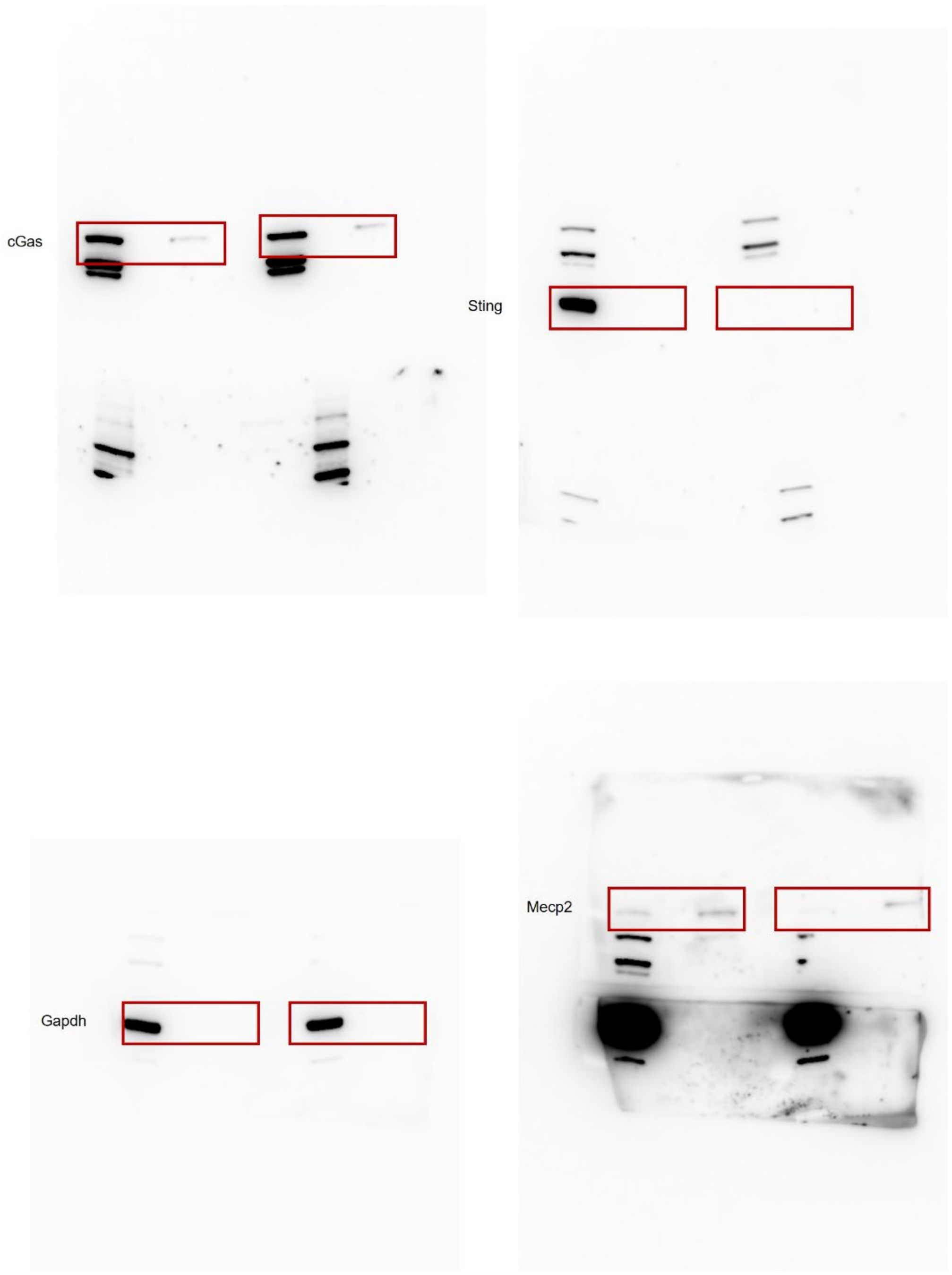

RAW data for Supplementary figure 3C.

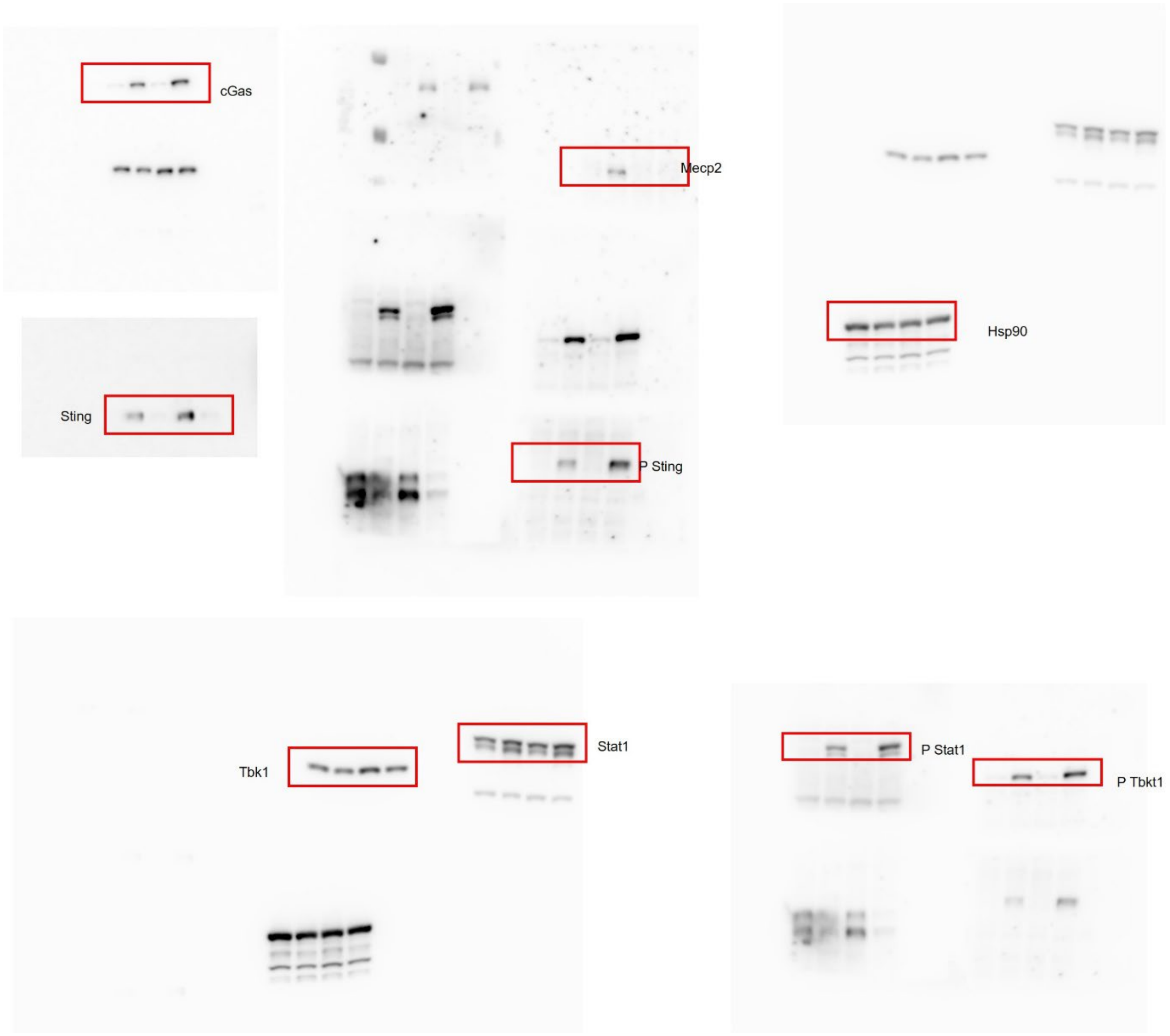

RAW data for Supplementary figure 4D.

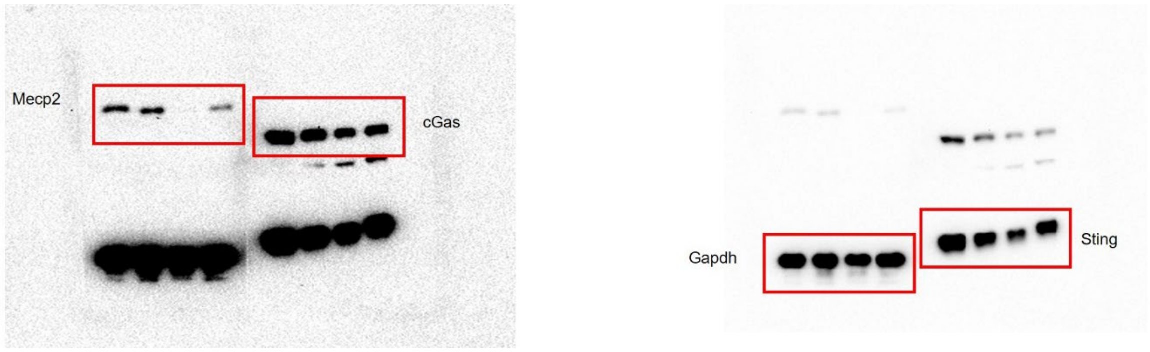

RAW data for Supplementary figure 4E.

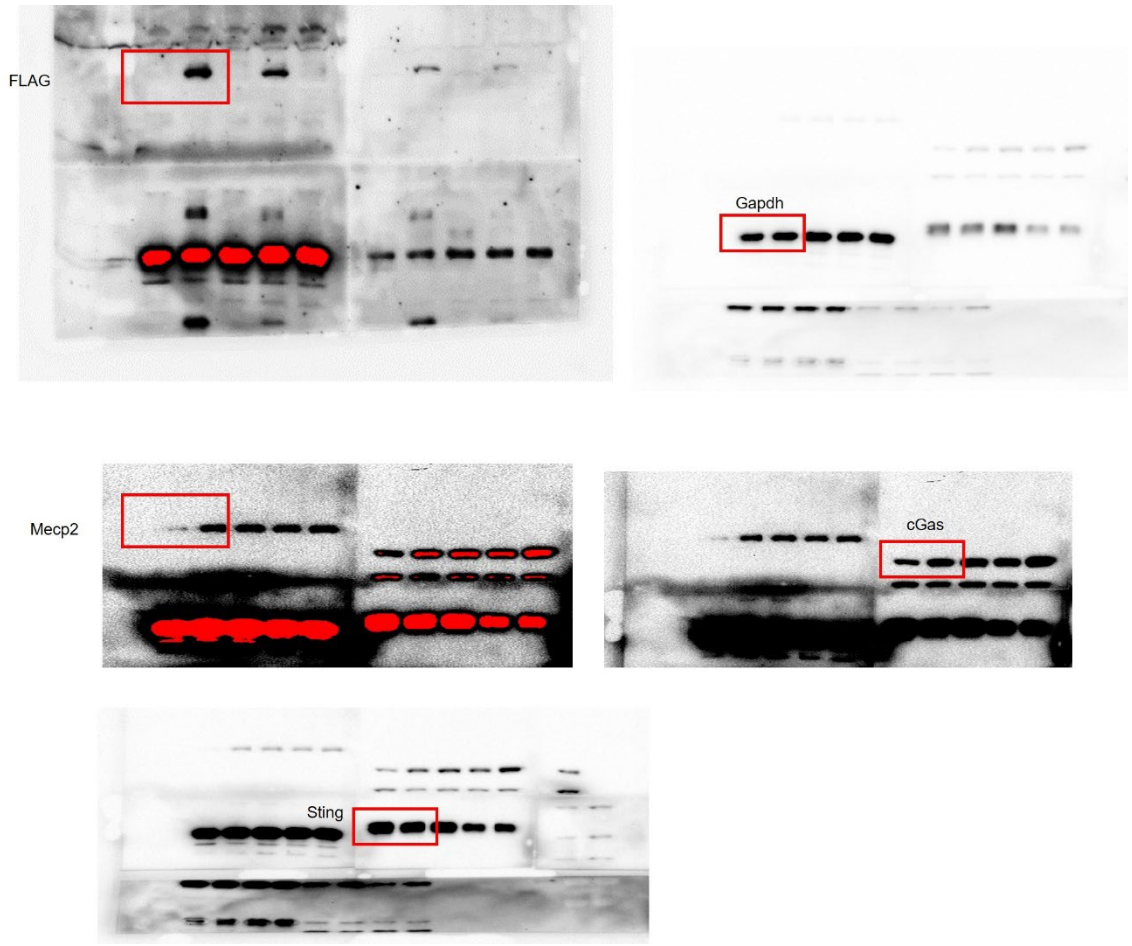

RAW data for Supplementary figure 4G.
