# Supplementary material for: The methyl-CpG-binding protein 2 inhibits cGAS-associated signaling
Source: Nat Commun. 2025 Nov 7;16:9827. doi: 10.1038/s41467-025-65713-z (PMC12595089; doi:10.1038/s41467-025-65713-z)
Supplement: Supplementary file 2 — Reporting Summary [file 41467_2025_65713_MOESM2_ESM.pdf]

nature portfolio

Corresponding author(s): Nadine Laguette

Last updated by author(s): Jun 27, 2025

Reporting Summary

Nature Portfolio wishes to improve the reproducibility of the work that we publish. This form provides structure for consistency and transparency in reporting. For further information on Nature Portfolio policies, see our Editorial Policies and the Editorial Policy Checklist.

Statistics

For all statistical analyses, confirm that the following items are present in the figure legend, table legend, main text, or Methods section.

n/a	Confirmed	
☐	☒	The exact sample size (*n*) for each experimental group/condition, given as a discrete number and unit of measurement
☐	☒	A statement on whether measurements were taken from distinct samples or whether the same sample was measured repeatedly
☐	☒	The statistical test(s) used AND whether they are one- or two-sided<br>*Only common tests should be described solely by name; describe more complex techniques in the Methods section.*
☒	☐	A description of all covariates tested
☒	☐	A description of any assumptions or corrections, such as tests of normality and adjustment for multiple comparisons
☐	☒	A full description of the statistical parameters including central tendency (e.g. means) or other basic estimates (e.g. regression coefficient) AND variation (e.g. standard deviation) or associated estimates of uncertainty (e.g. confidence intervals)
☐	☒	For null hypothesis testing, the test statistic (e.g. F, t, r) with confidence intervals, effect sizes, degrees of freedom and P value noted<br>*Give P values as exact values whenever suitable.*
☒	☐	For Bayesian analysis, information on the choice of priors and Markov chain Monte Carlo settings
☒	☐	For hierarchical and complex designs, identification of the appropriate level for tests and full reporting of outcomes
☐	☒	Estimates of effect sizes (e.g. Cohen's d, Pearson's r), indicating how they were calculated

Our web collection on statistics for biologists contains articles on many of the points above.

Software and code

Policy information about availability of computer code

Data collection	*Provide a description of all commercial, open source and custom code used to collect the data in this study, specifying the version used OR state that no software was used.*
Data analysis	All used softwares are mentioned in the manuscript.

For manuscripts utilizing custom algorithms or software that are central to the research but not yet described in published literature, software must be made available to editors and reviewers. We strongly encourage code deposition in a community repository (e.g. GitHub). See the Nature Portfolio guidelines for submitting code & software for further information.

Data

Policy information about availability of data

All manuscripts must include a data availability statement. This statement should provide the following information, where applicable:

- Accession codes, unique identifiers, or web links for publicly available datasets
- A description of any restrictions on data availability
- For clinical datasets or third party data, please ensure that the statement adheres to our policy

The sequencing data that support the findings of this study are available in Gene expression omnibus (GEO) with the accession number: GSE286551. Other source data for figure 1-6 and S1-S6 are provided with the paper.

Research involving human participants, their data, or biological material

Policy information about studies with human participants or human data. See also policy information about sex, gender (identity/presentation), and sexual orientation and race, ethnicity and racism.

Reporting on sex and gender	No human participant was enrolled for the present study.
Reporting on race, ethnicity, or other socially relevant groupings	No human participant was enrolled for the present study.
Population characteristics	No human participant was enrolled for the present study.
Recruitment	No human participant was enrolled for the present study.
Ethics oversight	No human participant was enrolled for the present study.

Note that full information on the approval of the study protocol must also be provided in the manuscript.

Field-specific reporting

Please select the one below that is the best fit for your research. If you are not sure, read the appropriate sections before making your selection.

[x] Life sciences [ ] Behavioural & social sciences [ ] Ecological, evolutionary & environmental sciences

For a reference copy of the document with all sections, see nature.com/documents/nr-reporting-summary-flat.pdf

Life sciences study design

All studies must disclose on these points even when the disclosure is negative.

Sample size	Experiments were repeated a minimum of 3 times, in triplicates.
Data exclusions	No data was excluded.
Replication	Experiments were repreated independently by lab members and outside the lab, ensuring their reproducibility.
Randomization	Experiments reported herein were mostly in vitro biochemistry, molecular and cell biology approaches. No randomization was performed. In vivo data were postmortem analyses on mice of different genotypes, where mice were randomized prior to sacrifice.
Blinding	Experiments reported herein were mostly in vitro biochemistry, molecular and cell biology approaches. No blinding was performed. In vivo data were postmortem analyses on mice of different genotypes. Blinded analyses were performed.

Reporting for specific materials, systems and methods

We require information from authors about some types of materials, experimental systems and methods used in many studies. Here, indicate whether each material, system or method listed is relevant to your study. If you are not sure if a list item applies to your research, read the appropriate section before selecting a response.

Materials & experimental systems

n/a	Involved in the study	
[ ]	[x]	Antibodies
[ ]	[x]	Eukaryotic cell lines
[x]	[ ]	Palaeontology and archaeology
[ ]	[x]	Animals and other organisms
[x]	[ ]	Clinical data
[x]	[ ]	Dual use research of concern
[x]	[ ]	Plants

Methods

n/a	Involved in the study	
[x]	[ ]	ChIP-seq
[x]	[ ]	Flow cytometry
[x]	[ ]	MRI-based neuroimaging

Antibodies

Antibodies used	For WB, Primary primary antibodies used include: anti-Mecp2 (1:500; Cell signaling D4F3, # 3456T), mouse-specific anti-cGas (1:1000; Cell signaling D3080, # 31659S), anti-phosphorylated Irf3 (1:500; Cell Signaling 4D4G, # 4947S), anti-Irf3 (1:1000; Cell Signaling D83B9,#)4302), anti-phosphorylated Tbk1 (1:1000; Cell Signaling D52C2, # 5483S), anti-Tbk1 (1:1000; Cell Signaling D1B4, 3 3504S), anti-Sting (1:1000; Cell Signaling D2P2F, # 13647S), anti-phosphorylated Sting (1:1000; Cell Signaling D8F4W, # S365), anti-Hsp90 (1:1000; Cell Signaling C45G5, #4877), anti-Gapdh (1:5000; Proteintech Europe # 60004-1-Ig), anti-Lamin B1 (1:1000, Santa Cruz Biotechnology # sc-374015), anti-Acetylated Histone H3 (1:1000; Santa Cruz Biotechnology # sc-56616), anti-Flag (1:1000; Sigma #

F1804), anti-Tubulin α (# 66031-1-Ig, Proteintech Europe, 1:10,000), and anti-Ranbp1 (1:100; Santa Cruz Biotechnology # sc-374352). Secondary antibodies used were Membranes were incubated with Horseradish peroxidase (HRP)-coupled secondary antibodies (Cell Signaling, anti-rabbit # 7074, anti-mouse # 7076) at 1:2000 dilution for 1 hour at room temperature.

For immunofluorescence, primary antibodies used are: anti-Mecp2 (Cell signaling D4F3, # 3456T) used at 1:50 dilution, anti-cGas (Cell signaling D3080, # 31659) used at 1:100 dilution, anti-IRF3 (Cell signaling D83B9 ,#4302S) used at 1:100 dilution, anti-Ranbp1 (Santa Cruz Biotechnology # sc-374352) used at 1:50 dilution, anti-dsDNA (Abcam # ab27156) used at 1:100 dilution, and anti-GFP (Abcam # ab290) used at 1:100 dilution. Secondary antibodies were used at 1:200: Alexa Fluor 488 coupled goat anti-Rabbit IgG, (Thermofischer #R37116), Alexa Fluor 488 goat anti-Mouse IgG, (#A11001, Thermofischer), Alexa Fluor 594-coupled goat anti-Mouse IgG (Thermofischer #R37121), and Alexa Fluor 594-coupled goat anti-Rabbit IgG (#R37117, Thermofischer).

Validation

Commercial antibodies were used. Key antibodies were validated on in house knockout cell lines (cGas, Mecp2, Sting).

Eukaryotic cell lines

Policy information about cell lines and Sex and Gender in Research

Cell line source(s)

Wild-type (WT) murine embryonic fibroblast (MEF) and cGAS-deficient MEF (MEFcGas-/-) were a gift of S. R. Paludan. Trex1-deficient MEF (MEFTrex1-/-) were a gift from J. Rewhinkel. BHK21 cells were a gift of Olivier Moncorgé (IRIM, Montpellier). Parental RAW246RAW264.7 (Mus musculus), NIH3T3 (Mus musculus), HEK293T (Homo sapiens) and THP-1 (Homo sapiens) cells were obtained from the American Type Culture Collection (ATCC).

Authentication

None of the cell lines used was authenticated.

Mycoplasma contamination

All cell lines tested negative for mycoplasma contamination.

Commonly misidentified lines
(See ICLAC register)

No commonly misindentified lines was used in the study.

Animals and other research organisms

Policy information about studies involving animals; ARRIVE guidelines recommended for reporting animal research, and Sex and Gender in Research

Laboratory animals

Post mortem samples from Mecp2-deficient (Jackson (B6.129P2(c)- Mecp2tm1-1Bird) male mice were a kind gift from Emmanuel Valjent (IGF Montpellier) and Adrian Bird (University of Edinburgh).

Wild animals

No wild animal was used int he study.

Reporting on sex

Male mice were used because the gene of interest (Mecp2) is X-linked.

Field-collected samples

No sample was collected from the field.

Ethics oversight

Only post mortem samples were used, which are not subjected to ethical regulation.

Note that full information on the approval of the study protocol must also be provided in the manuscript.

Plants

Seed stocks

No plant was used in the study.

Novel plant genotypes

No plant was used in the study.

Authentication

No plant was used in the study.
